# Health, access and nutritional issues among low-income population in Malaysia: introductory note

**DOI:** 10.1186/s12889-019-6852-8

**Published:** 2019-06-13

**Authors:** Suzana Shahar, Huijin Lau, Sharifa Ezat Wan Puteh, Sofia Amara, Norizan Abdul Razak

**Affiliations:** 1 Centre for Healthy Aging and Wellness, Faculty of Health Sciences, Universiti Kebangsaan Malaysia, Jalan Raja Muda A. Aziz, 50300 Kuala Lumpur, Malaysia; 2 Department of Community Health, Faculty of Medicine, Universiti Kebangsaan Malaysia, Jalan Yaacob Latif, Bandar Tun Razak, 56000 Cheras, Kuala Lumpur, Malaysia; 3 International Life Sciences Institute (ILSI) Southeast Asia Region, 18 Mohamed Sultan Road #03-01, Singapore, 238967 Singapore; 4 Women Leadership Centre, Universiti Kebangsaan Malaysia, 43000 Bangi, Selangor Malaysia

**Keywords:** Low-income group, Malaysia, Health equalities, Nutrition, Quality of life

## Abstract

The current issue of BMC Public Health presents work by the Consortium of Low Income Population Research (CB40R), highlighting a comprehensive aspect of health, i.e., physical health, mental health, health behaviour and health financing; and also nutrition involving all stages of lifespan of the socioeconomic deprived group in Malaysia.

Consortium of B40 Research (CB40R) reposited and harmonised shared, non-identifiable data from epidemiological studies involving low income population (B40) in Malaysia. CB40R also performed joint or mega-analyses using combined, harmonised data sets that yield collated results with enhanced statistical power, more variabilities (study population, geographical regions, ethnicities and sociocultural groups) to better understand the needs, characteristics and issues of B40 groups in Malaysia. It also aimed to develope a system/framework of minimum/standard variables to be collected in research involving B40 in future. For this special issues, members of the consortium have been invited to contribute an original article involving analysis of the health aspects, access to health and nutritional issues of the B40 samples.

All the papers in this special issue have successfully highlighted the health and nutritional issues (i.e., non-communicable disease (NCD), inflammatory bowel disease (IBD), knowledge towards sexually transmitted disease (STD), low birth weight, Motoric Cognitive Risk (MCR) syndrome, urinary incontinence), mental health, oral health and inequalities among the low-income group in Malaysia, including the rural population and also the urban poor. The low-income population in Malaysia is also at risk of both under- and over nutrition, of which specific cost effective strategies are indeed needed to improve their quality of life.

The low income population in Malaysia is facing various health challenges, particularly related to NCD and poor mental health, nutritional and physical function. There is a need for a sustainable intervention model to tackle the issues. It is also important to highlight that reducing SES disparities in health will require policy initiatives addressing the components of socioeconomic status (income, education, and occupation) as well as the pathways by which these affect health.

## Introduction

Malaysia aspires to be a developed nation by the year 2020 and if these development ambitions are to be attained Malaysia needs to reexamine its past approaches to reduce poverty and inequalities in the country. In line with the Sustainable Goal theme ‘no one will be left behind’, efforts have been planned or executed to ensure Malaysians get access to the wealth and development of the nation. However, a substantial proportion of the population is still struggling to achieve socioeconomic and health equalities, i.e., the low-income groups. Current scenario indicates that Malaysia is no longer just grappling with absolute poverty but also with relative poverty, pockets of persistent poverty, the traditional rural poverty, and urban poverty as well as increasing inequalities [1]. During the Tenth Malaysia Plan (10MP 2011–2015), the bottom 40 (B40) consists of 2.4 million households, with 73% bumiputeras (locals) and the remaining 27% non-bumiputeras (non-locals). Whilst in the 11MP (2016–2020), there are 2.7 million households of B40 (mean monthly income of RM2, 537.00), with 68% bumiputeras (locals) and 32% non-bumiputeras (non-locals); and 56% in urban and 44% in rural areas [2]. Socioeconomic problems, health and food insecurity continue to be the problems of the low income group or B40 [2, 3]. A number of strategic plans and policies targeted for the B40 groups have been implemented by various government agencies and corporate sectors through its Corporate Social Responsibility (CSR) programme. However, relatively little empirical data exists in assessing its effectiveness. A recent publication examining such programme among farmers indicated that both CSR and government support had a significant effect on the targeted constructs. However, the government support produces negative impact on income creation, thus, CSR has a more important role in the future [4]. Such, formative evaluation studies are essential to further assess the cost effectiveness of the current programmes for the B40 or low-income groups. Further, it is also a concern whether adequate or relevant need assessment or situational analysis has been conducted prior to planning programmes or allocating resources to alleviate the quality of life or living condition of the low income group. Issues and problems faced by this group of the population have received the attention of not just policy makers, strategists, community leaders, politicians and also the corporate; but also researchers and scientists. However, it is observed that for the past few years, research among this group has mainly focused on descriptive analysis, mostly qualitative, involving single discipline and small sample sizes and also using secondary data. Recognising this gap, an effort is being embarked on formation of Consortium of B40 Research (CB40R), with the following aims:

1. To form CB40R to reposit, harmonise shared, non-identifiable data from epidemiological studies involving B40 sample in Malaysia;
2. To perform joint or mega-analyses using combined, harmonised data sets that yield collated results with enhanced statistical power, more variabilities (study population, geographical regions, ethnicities and sociocultural groups) to better understand the needs, characteristics and issues of B40 groups in Malaysia;
3. To develop a system/ framework of minimum/ standard variables to be collected in research involving B40 in future.

Socioeconomic status (SES) underlies three major determinants of health: health care, environmental exposure, and health behavior. In addition, chronic stress associated with lower SES may also increase morbidity and mortality [5]. Thus, the current issue of BMC Public Health presents work by the CB40R, highlighting a comprehensive aspect of health, i.e., physical health, mental health, health behaviour, literarcy and financing; and also nutrition involving all stages of lifespan. The paper by Puteh et al. [6] opens this special issue by analysing the quality of life of the low-income population. Using a semi-guided self-administered questionnaire, they found that being single, had low household income, and presence of chronic medical illness were associated with low quality of life. The findings are important for the purpose of improving well-being and health status especially among the low SES population. Low SES in rapidly developing countries such as Malaysia is not just associated with conventional infectious diseases. Non-communicable diseases (NCD) has also emerged as a health threat as presented in articles by Harris et al. [7] among low income adults in rural coastal communities in Sabah, and by Norafidah et al. [8] among women. Obesity, a predisposing factor for NCD, has been observed in 22.8% of Malaysian children from the low-income group. As reported by Poh et al. [9], it is very much associated with poor cognitive function. Another important risk factor of NCD is smoking, now seen as common among Malaysian adolescents from the low-income group, as presented by Nur Atikah et al. [10]. Unhealthy lifestyle practices by Malaysians, particularly the low-income population, contribute to an increased risk of NCD including inflammatory bowel diseases (IBD), as presented by Mohktar et al. [11]. In fact, the occurrence of IBD in Malaysia is now similar to the figures commonly seen in developed countries. Chronic stress is one of the factors influencing IBD. Stress and mental health problems have been reported to affect one third of Malaysian population [12]. The low-income group has been identified as vulnerable towards mental health problems, as shown by the occurrence of workplace bullying and psychological distress among employees from this group [13]. They are also more likely to be absent from work due to lower income, divorce or separation, chronic diseases such as kidney disease, diabetes and migraine [14]. Mental health issues should be tackled early even in the younger population such as the adolescents. This special issue presents important work on mental health among adolescents from the low-income groups by Ibrahim et al. [15, 16]. Apparently, there is still lack of social support to address increased suicide ideation among marginalised adolescents in Malaysia, with those in the younger age group less likely to seek advice from mental health professionals. This reluctance is due to fear of social stigma when one is being diagnosed as having mental health problems and also due to poor health literacy. In a study by Mohamad Zin et al. [17], young adults were found to have poor health knowledge, attitude and practice towards sexually transmitted diseases. This would increase risk of infectious diseases and malnutrition. Pockets of malnutrition still exist, particularly among women from the low-income group that increases risk of low birth weight, as presented by Kau et al. [18]. Among indigenous school children in Malaysia, vision impairment is a health concern [19].

Malnutrition and inadequate diet are factors associated with low socioeconomic status among older adults, as has been reported by Shahar et al. [20]. Food consumption is related to ability to eat, prepare and purchase food. Rosli et al. [21] in their work among older adults in a rural area of Malaysia highlighted the association between oral health and nutritional status. Mental health problems are also prevalent among older adults. Baharudin et al. [22] stressed the importance of recognising behavioral-psychological symptoms of dementia (BPSD) among older adults. A study by Lau et al. [23] highlighted new geriatric syndromes emerging among low-income older adults, ie. Motoric Cognitive Risk syndrome, as a result of subjective memory complaints and slow gait. Whilst, Murukesu et al. [24] presented interesting findings on the occurrence and risk factors of the well-established geriatric syndrome, i.e., urinary incontinence among Malaysian older adults according to SES in both urban and rural areas.

### Income disparity among low SES

Inadequate income has always been an obstacle for the low-income population in getting access towards essential health care, conducive living including adequate nutrition. Aizuddin et al. [25] examined health financial model among the extreme condition, ie. the urban homeless. A willingness to pay (WTP) study, examined patient’s willingness to pay treatment cost, through out of pocket (OOP) payment for acute or chronic diseases [26]. Respondents were sampled among out patients attending public health care facilities in an urbanized, semi urban setting. Majority of respondents were young, female, of lower education and lower income. A total of 234 respondents (72.2%) were not willing to pay for drug charges. WTP for drugs either for chronic or acute illness were low at median value of MYR10 per visit (an average of USD 3.8 per visit). However, the actual providers cost for treatment, would generally be at between MYR40–50 per visit. Bivariate analysis showed that lower numbers of dependent children (< 3), higher personal and household income are associated with higher WTP. Multivariate analysis showed only number of dependent children (< 3) as significant (*p* = 0.009, 95% CI 1.27–5.44) predictor to drugs’ WTP.

Access to essential treatment also showed disparity in health outcomes. An example of this is cancer. Cancer is one of the major causes of Disability Adjusted Life Years (DALYs) in the country. Cancer is also a major health burden to low-income groups in Malaysia [27]. The five common cancers among males were cancers of the colorectal (16.3%), lung (15.8%), nasopharynx (8.1%), lymphoma (6.8%) and prostate (6.7%). Among females, the five most common were cancers of the breast (32.1%), colorectal (10.7%), cervix uteri (7.7%), ovary (6.1%) and lung (5.6%) [28].

Protocols needed to detect cancer include mammogram test (against breast cancer), Pap smear (against cervical cancer), chest X ray (against lung cancer) and stool occult blood test (against colorectal cancer). These can be readily accessed in most public hospitals for nominal payment. However, low and inaccurate knowledge in early signs and symptoms may impede individuals’ health seeking behavior and thus, treatment. Generally the stages of cancer, ranges (increasing in severity), from stage I (least invasive), II, III and most severe with metastatic spread, would be stage IV. It has been found in a few studies [29, 30] that most inpatients in late stages (stage III and IV) are among the low-income group. Hence, this shows that low-income individuals have higher risk towards catastrophic health expenditure [29], receive less cancer screening access and possibly late detection.

Methods have been developed to improve the ability of low-income groups to consume a balanced diet, in spite of financial constraints. Alaini et al. [31] presented the linear programming method to plan for a balanced diet when there is a limitation in income. They used this approach to develop a Low Cost Cancer Prevention Food Plan for adults in the metropolitan city of Kuala Lumpur, in which food prices and cost of living were considered in order to meet the dietary needs of a low-income population.

## Conclusion

All the papers in this special issue have successfully highlighted the health issues and inequalities among the low-income group in Malaysia, including the rural population and also the urban poor. The low-income population in Malaysia is at risk of both under- and over nutrition, of which specific cost effective strategies are indeed needed to improve their quality of life. Reducing SES disparities in health will require policy initiatives addressing the components of socioeconomic status (income, education, and occupation) as well as the pathways by which these affect health.

## Abbreviations

B40: Bottom 40
BPSD: Behavioral-psychological symptoms of dementia
CB40R: Consortium of B40 Research
CSR: Corporate Social Responsibility
DALYs: Disability Adjusted Life Years
IBD: Inflammatory bowel disease
MP: Malaysia Plan
NCD: Non-communicable disease
OOP: Out of pocket
SES: Socioeconomic status
WTP: Willing to pay
